# The Application of Sector-Scanning Sonar: Strategy for Efficient and Precise Sector-Scanning Using Freedom of Underwater Walking Robot in Shallow Water

**DOI:** 10.3390/s20133654

**Published:** 2020-06-29

**Authors:** Hyuk Baek, Bong-Huan Jun, Myounggyu D. Noh

**Affiliations:** 1 Ocean System Engineering Research Division, KRISO, 32 Yuseong-daero, 1312 beon-gil, Yuseong-gu, Daejeon 34103, Korea; bhjeon@kriso.re.kr; 2 Department of Mechatronics Engineering, Chungnam National University, 99 daehak-ro, Yuseong-gu, Daejeon 34134, Korea; mnoh@cnu.ac.kr

**Keywords:** sector scanning sonar, CRABSTER, CR200, underwater walking robot, ROV, reverberation

## Abstract

In this paper, we discuss underwater walking robot technology to improve the quality of raw data in sector-scanning sonar images. We propose a strategy for an efficient and precise sector-scanning sonar image acquisition method for use in shallow, strong tidal water with a curved and sloped seabed environment. We verified the strategy by analyzing images acquired through a sea trial using the sector-scanning sonar installed on the CRABSTER (CR200). Before creating this strategy, an experiment was conducted to acquire the seabed image near a pier using a tripod and vertical pole. To overcome the problems and limitations revealed through image analysis, we established two technical strategies. In conclusion, we were able to achieve those technical strategies by using the CR200, which is resistant to strong current, and its six legs provide freedom of movement, allowing for a good sonar attitude.

## 1. Introduction

While visibility can range from 6 to 15 m in the deep ocean, this range can be reduced to 1 to 6 m in near-shore water. In ports, estuaries, and other human-occupied waters, visibility can be less than 1 m [1]. The SONAR (sound navigation and ranging) system is the most widely adopted solution for remote sensing and is very useful for underwater observation and surveillance in coastal waters with poor visibility due to sediments [2,3,4,5]. Sector-scanning sonars are a device used to capture two-dimensional images and are widely used for vehicle navigation, obstacle avoidance, and general inspectional surveys of the surrounding environment by unmanned underwater vehicles (UUV) [6,7,8].

A scanning sonar utilizes reverberation to detect targets. The reverberation can occur from the volume of the sea (volume reverberation), from the sea surface (sea surface reverberation), and the sea bottom surface (sea bottom reverberation) [9,10,11]. These three types of reverberation signals are indistinguishable due to the randomness in the signal. As the level of reverberation decreases with range, the rate of positioning errors increases, which can decrease the performance of in-vehicle navigation. Because of this navigation deficiency, more robust object detection and additional information regarding object movement are necessary to ensure obstacle avoidance. In some cases, special equipment must be added to capture accurate images of the surroundings [1,12,13].

Extracting useful information from reverberation signals is an important issue in sonar signal processing. Since the reverberation is correlated with the reflection from the target, it is necessary to remove the reverberation or to increase the signal level to efficiently detect the reflection from the target. There are many algorithms to improve detection where reverberation exists. Karine et al. [14] proposed a space–time adaptive processing method for low-frequency sonar. Song et al. [15,16,17] used a time-reversal mirror for reverberation nulling to cancel the reverberation. Guillaume et al. [18,19] suggested an algorithm using principal component inverse (PCI) algorithm to improve the detection in the presence of reverberation. Past research exists regarding blind source separation (BSS) technology, including Cong et al.’s [20] presentation of a Bean-space-based BSS algorithm to improve target echo. Research efforts to increase the reflection signal strength were also carried out. Xu et al. [21] attempted to increase the strength of the reflection signal by using a sliding window. Although the research to separate the target reflection signal from echo improved the detection capability to some extent, it focuses on post-processing the signal rather than enhancing the quality of raw data. If we can capture high-resolution raw data, the detection capability can be significantly improved.

Atherton [22] claimed that any method to enhance the raw data quality in shallow water, regions with strong currents, and an uneven or sloped sea bottom must consider the following five points:

- Lessens surface multipath;
- Reduces surface reverberation;
- Head stability;
- Head position and alignment;
- Decreases slant range distortion.

Current techniques to improve the raw data while satisfying the points above include using a narrower vertical beam or tilt transducer, and installing the sonar on a drop mount, pole mount, or spreader bar; however, the sensors and equipment must be adapted to each situation. Continuous acquisitions of images require accurately moving the sensor, which is very difficult in practice.

To overcome the limitations of the current methods and to improve the quality of raw data, in this paper, we propose two technical methods (hula hoop motion (HHM) and control posture) to be used with new techniques using an underwater walking robot: the CRABSTER (CR200). 

## 2. Limitations of Existing Methods

We analyzed the acquired sonar image from the HEMIRE [23,24] remotely operated vehicle (ROV) operation in turbid water in 2010 and the vertical/horizontal image acquired by tripod and pole-mount deployment from the sonar test conducted at the pier of the South Sea Research Institute of KIOST (Korea Institute of Ocean Science and Technology), collected from 3 April to 6 April 2012, to determine the limitations of sonar operation.

### 2.1. Specifications of Sector-Scanning Sonar

**Figure 1:**
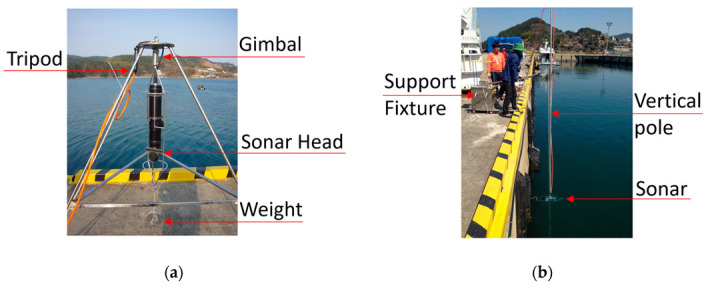
General sector-scanning sonar image acquisition methods: (**a**) Horizontal image acquired by tripod deployment; (**b**) Vertical image acquired by pole-mount deployment.

**Figure 2:**
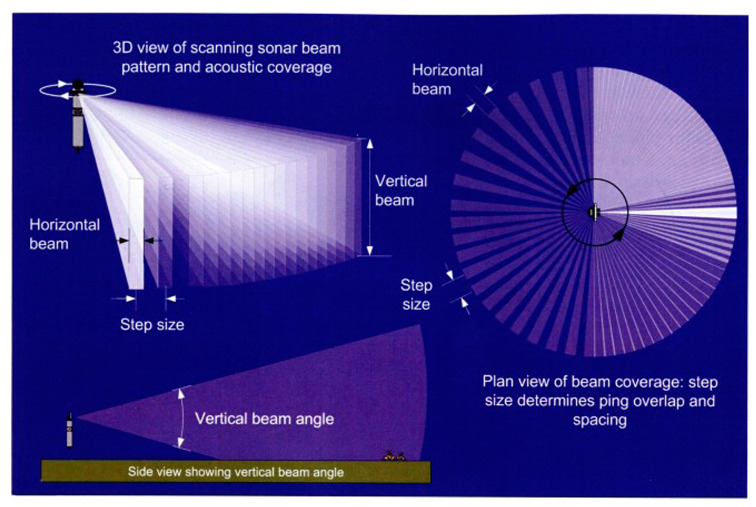
Sector-scanning sonar acoustic coverage [22].

Figure 1 shows the operational equipment for sector-scanning sonar. The sector-scanning sonar used in this research was a high-resolution scanning sonar made by Kongsberg Mesotech (Kongsberg, Canada). Table 1 lists the specifications and Figure 2 illustrates the acoustic coverage of the sonar [22]. It is operable up to a depth of 3000 m and uses a frequency of 675 kHz. It takes 36 seconds for a full 360° scan if the step size is 1.8° at 100 m range. Polar images were obtained by a sequential scan of 0.9° horizontal and 30° vertical beam width. It is assumed that the side lobes do not affect the horizontal image analysis.

### 2.2. Limitations of Existing Methods

#### 2.2.1. Multipath and Sea Surface Reverberation

**Figure 3:**
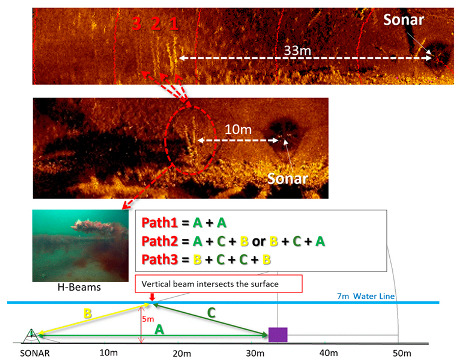
Multipath as seen in the sonar image.

Multipath noise and sea surface reverberation significantly affect the resolution when acquiring the images of a target at the seabed in shallow water [22]. Figure 3 demonstrates three possible paths that multipath noises can occur. When a thermocline is present, the number of paths can increase to five. In our field tests, the depth was about 7 m. The operation range of the sonar on the tripod was 50 m. The step size was 0.225°. The top image in Figure 3 shows the target located at 33 m from the sonar. The closest target from the sonar (target 1) is shown as a sonar image of a H-beam and has the strongest reflection signal. The images of targets 2 and 3 are less clear as the paths become longer compared to target 1. The middle image in Figure 3 was taken when the sonar was moved closer to the target (a distance of 10 m). Targets 2 and 3 are not shown here, which means that targets 2 and 3 in the top image are the results of multipath noise. The actual picture of the beam taken by a diver is shown at the left of the third row of Figure 3. The bottom plot of Figure 3 illustrates three possible paths. If the target is at the same level as the sonar, we can draw an isosceles triangle with the apex being the point where the vertical beam intersects the surface. This implies that the multipath noise can be eliminated if the apex is located at the halfway point of the sonar range.

**Figure 4:**
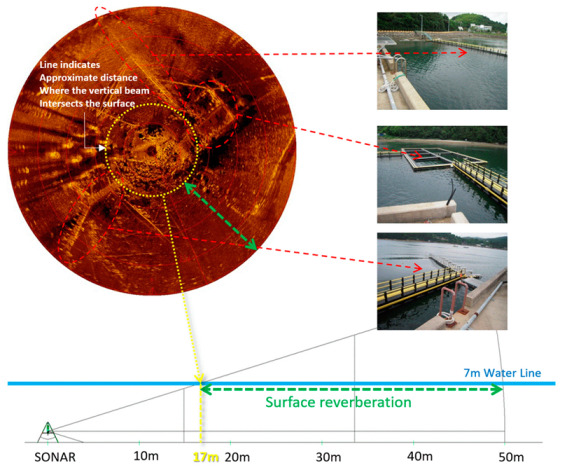
Influence of sea surface reverberation [25].

**Figure 5:**
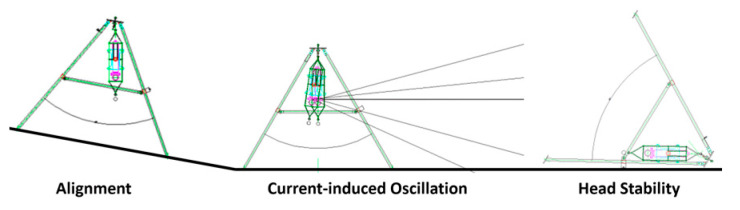
Disadvantages of the tripod with a gimbal in a strong current.

The sonar image in Figure 4 shows a representative case of sea surface reverberation. Again, the water depth was 7 m. The sonar operation range was 50 m with a step size of 0.225°. The green arrow on the sonar image indicates the at-risk region where sea surface reverberation can occur. To the right of the sonar image are the actual pictures of a fish-cultivation farm and a float bridge connecting the farm with the land. The farm and the bridge are shown as artifacts in the sonar image (marked by the dotted ellipses and arrows). The mechanism of surface reverberation is illustrated in the bottom drawing of Figure 5. The reverberation starts at approximately 17 m from the sonar. The structure above the surface is mirrored and superimposed with the underwater image due to the reverberation. 

Figure 3 and Figure 4 show the limitations in acquiring a high-resolution underwater image in shallow water due to multipath noise and surface reverberation. To overcome these limitations, several methods were attempted: multiple mapping at ranges where the surface reverberation and multipath noise do not occur, using a reduced vertical beam size, or scanning in the direction of the surface by tilting the sensor head [22]. It takes considerable time and effort to gather sonar data, and additional scanning sonars with a narrow vertical angle were required.

#### 2.2.2. Head Stability and Alignment

The following three techniques should be considered for acquiring high-resolution sonar images using a tripod in shallow water, regions with a strong current, or an uneven or sloped sea bottom. First, the sonar head must be stabilized by minimizing the current-induced oscillation. Second, the sonar must be properly aligned to provide the necessary posture (elevation, inclination angle, direction angle, etc.). Finally, the sonar must be arbitrarily located. In an environment with weak or no tidal current, it is possible to maintain the image quality even if the tripod is on an uneven or sloped seafloor because the sonar head is aligned with the gravitation due to the gimbal mechanism; however, as shown in Figure 5, it was difficult for us to install the sonar head in a stable position on a sloped floor parallel to the ground, and also not easy to suppress the oscillation due to the strong current. There are several ways to do achieve stability: by deploying sensors from a fixed structure on the surface or barge using a spreader bar, using a fixed tripod instead of the gimbal, and installing a stabilization fin to remove the vortex behind the sonar using a drop cable deployment in a strong current environment; however, it is difficult to maneuver a sonar and maintain a desired posture and position in an uneven or sloped sea bottom with a strong current, even if these methods are used.

#### 2.2.3. Slant Range Distortion

**Figure 6:**
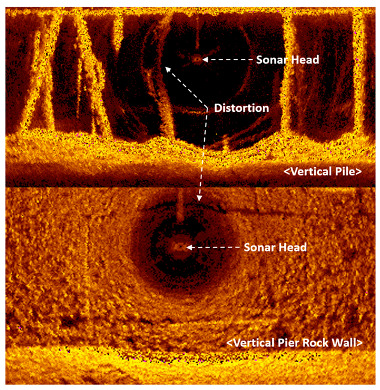
Slant range distortion.

Sector-scanning sonar is used to inspect slopes and vertical structures such as beams, dams, and bridges. For these applications, the sonar head is installed vertically, and close to the target surface. Both sector-scanning sonar and side-scanning sonar have a problem that creates an illusion of deformation of the object adjacent to the sonar, which is called slant range distortion. This is caused by displaying the 3D data on a 2D monitor, and is generated from the side lobe of the acoustic sensor [22]. The top image of Figure 6 shows an image of vertical piles supporting a pier, and shows the distortion that occurs in the pile near the sonar head. The image below shows a vertical pier rock wall and that the shadow of the stonework at 12 o’clock is curved from the sonar head. Conventionally, the problem is solved by overlapping the normal image acquired at a distance and removing the distorted image. 

#### 2.2.4. Sonar Operation with General ROV

**Figure 7:**
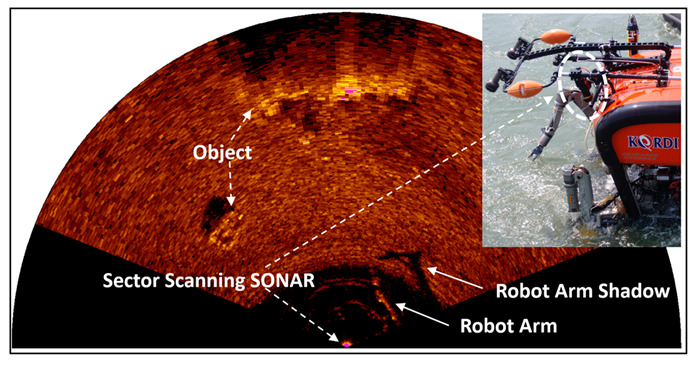
Sector-scanning sonar application of a work-class remotely operated vehicle (ROV).

If a sector-scanning sonar is usually installed on an underwater robot or vehicle, it is highly useful to detect objects, avoid obstacles, and acquire the necessary information for navigation [6]. By using the precision position sensor installed in a remotely operated vehicle (ROV), it is possible not only to know the position of the acquired image but also to continuously obtain the image by moving to the desired position. As shown in Figure 7, the work-class ROV has the advantage of estimating the pose of the manipulator and can maneuver it by observing the shadow of the manipulator; however, once the position of the sonar is fixed to the ROV frame, it is difficult to change the position during operation, and, in the case of a work-class ROV operation, an additional sonar is required to cover the full 360° due to its size. The most general issue is that the standard form of the ROV is a shape of a rectangular box, so it is hard to move the ROV along the desired course while also maintaining the desired heading and altitude under an environment with a strong current. 

### 2.3. Strategies to Overcome the Limitations of Conventional Sonar Operation Methods

Previous sections list the issues regarding reverberation and distortion due to the limitations of conventional sonar operation. To overcome these limitations, the environmental aspects such as shallow water, regions with a strong current, and an uneven or sloped sea bottom must be considered together. In this paper, we suggest strategies to improve the acquisition of high-resolution sonar images in the following way:

1. To continuously provide an inclined angle in a rotating sonar scanning direction;
2. To realize positioning, alignment, target tracking, and anti-oscillation by controlling the platform where the sonar is installed;
3. To acquire an efficient 360° omnidirectional image using a single sonar.

## 3. A New Concept of Sensor Operation Strategy

### 3.1. Seabed Image-Obtaining Strategies using an Underwater Walking Robot

**Figure 8:**
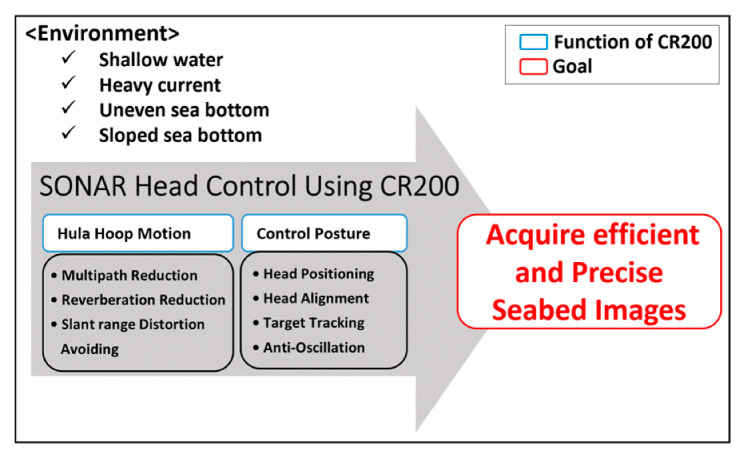
Two main functions of the CRABSTER (CR200) for the acquisition of efficient and precise seabed images.

Figure 8 shows a strategy for overcoming the conventional technical problems summarized in Section 2.3 using the CR200 under four environmental conditions (shallow water, regions with a strong current, and an uneven or sloped sea bottom) and efficiently obtaining a precise seabed image. HHM, expressed as a function of CR200, represents the technology corresponding to improvement 1 in Section 2.3. This technology selectively separates the sea surface reverberation from the bottom reverberation to reduce noise caused by both multipath and reverberation, and to minimize the slant range distortion that occurs when a support ship, a large structure, or an ice shelf are located close to the sonar, perpendicular to the up position. The second function of CR200, the control posture, is a technology that provides the desired posture and position to the sonar. As described in improvement 2 in Section 2.3, with this technology, the oscillation of the sonar can be suppressed and the ROV can move to the desired course while maintaining a certain altitude and heading. The last technology, described in improvement 3 in Section 2.3, maximizes the efficiency of image acquisition. This enables the sonar installed on the ROV to acquire the image of the seafloor of the widest area in one scanning, while minimizing the shadow section caused by the interference of the ROV body itself.

### 3.2. Introduction of Multi-Legged Underwater Robot CR200

The CR200 was developed in 2013 for exploration and scientific research on the seabed in areas where the current is very strong, such as the West Sea in Korea [26]. It is designed to investigate the seabed in detail and to be suitable for precise underwater work, featuring six legs, which consist of multiple joints instead of a propeller type actuator. The six legs consist of four dedicated legs with four degrees of freedom and two robotic arms with seven degrees of freedom, allowing it to walk on the seafloor like a crab and perform underwater work using its arms.

The specifications of the CR200 are as follows: it is 2.42 m long, 2.45 m wide, and 1.2 m high; it can lift its own body by 1 meter. The total weight is 650 kg; it can be operated up to a water depth of 200 m; the input power is 150 to 190 VDC (voltage direct current); the body structure of the CR200 has a streamlined form to minimize the effect of the current [27]. Table 2 shows the mechanical specifications of the CR200.

**Figure 9:**
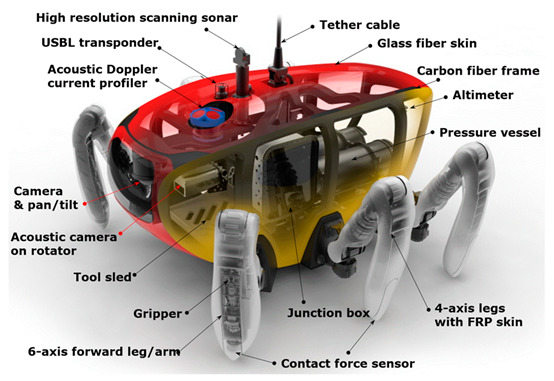
Equipment configuration of CR200.

The body frame of the CR200 and the composition of the equipment mounted inside are shown in Figure 9. It is equipped with an attitude and heading reference system (AHRS), a gyrocompass for measuring posture and a tool sled to be used as storage for various subsea tooling and sample collection. The equipment used for the underwater operation was an acoustic doppler current profiler (ADCP), which measures the direction and speed of current for a specific water layer, an ultra-short baseline (USBL) responder, which was used to measure the position of the robot, and several optical cameras for optical image acquisition. An acoustic camera for identifying objects within 10 m in a turbulent environment around the robot was mounted in the center of the body and a sector-scanning sonar that investigates a relatively large area was installed at the top to make it easy to acquire a 360° image. 

### 3.3. The Function of Control Posture

**Figure 10:**
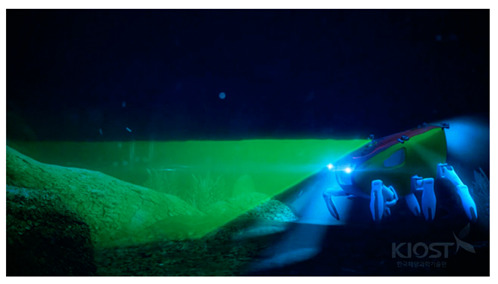
Tilt angle adjustment to the sector-scanning sonar concept for the CR200 [25].

Control posture is a technology that provides the required posture and position for the sensors, and was covered in detail in previous research, so in this paper, we lightly introduce the topic. There were previous studies on the “posture and walking control method of CR200 for precise underwater exploration using acoustic instrument” [28], “CR200 and an acoustic camera for survey of underwater cultural heritage” [29], and “Head alignment of a single-beam scanning sonar installed on a CR200” [30], and we demonstrated the capability of this technology through studies. Figure 10 shows the concept image of how the CR200 applies a tilt angle to the sector-scanning sonar.

### 3.4. Controlling of the HHM Inclination Angle

**Figure 11:**
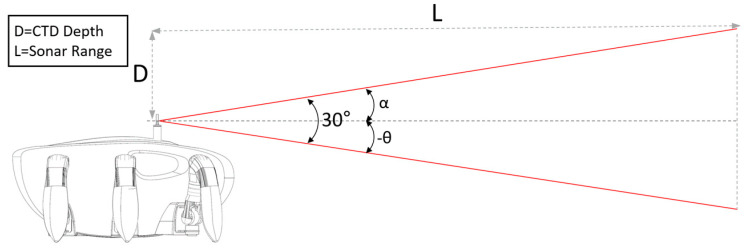
Hula hoop motion (HHM) inclination angle of the scanning sonar.

According to reference [25], the pitch angle, α, can be calculated by Equation (1), where D is a constant obtained by subtracting the altitude of CR200 from the water depth; this value can also be taken from the conductivity-temperature-depth (CTD) sensor. As shown in Figure 11, the vertical beam angle of the sensor is 30°, and as described in Section 2.2, the length is 25 m, which is useful to minimize noise as a result of multipath when the sonar range is 50 m. According to the equation, the α value can be calculated to avoid noise caused by multipath, which, depending on the change of the water depth and the resulting tilting angle, can be given to sonar and used as an input to the HHM inclination angle.

(1) $$\alpha =\tan^{-1}\frac{D}{L}$$

### 3.5. Kinematic Model of the CR200

**Figure 12:**
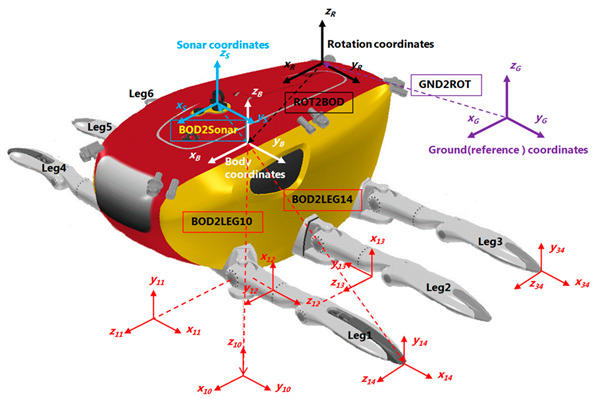
The coordinate system of the CR200 and installation of the scanning sonar [32].

The coordinate system of CR200 is shown in Figure 12. The position vector of the end of the leg relative to the center of the body ($P_{Li4}=\left\{\begin{array}{ccc} x_{Li4} & y_{Li4} & z_{Li4} \end{array}\right\}$) to generate the desired displacement of the body position ($dp_{B}=\left\{\begin{array}{cccc} dx & dy & dz & \begin{array}{cc} d\varphi & \begin{array}{cc} d\chi & d\phi \end{array} \end{array} \end{array}\right\}$) can be obtained by the following equation [28]:

(2) $${}^{B}T_{i4}={\left({}^{G}T_{B}^{p}{\;}^{B}T_{R}^{init}\;R_{ypr}{\left({}^{B}T_{R}^{init}\right)}^{-1}\right)}^{-1}{\;}^{G}T_{B}^{init}{\;}^{B}T_{i4}^{init}$$

where ${}^{B}T_{i4}$ is the transformation matrix from the body coordinate system to the end of the i-th leg coordinate system; ${}^{G}T_{B}^{p}$ is a transformation matrix that takes into account the positional displacement (${}^{G}p_{B}$) of the body-centered coordinate system viewed from a fixed coordinate system during posture transformation; ${}^{R}T_{B}$ is a transformation matrix from the rotation coordinate system to the body coordinate system considering the rotation transformation ($R_{ypr}$) defined in the rotation coordinate system during the change of the body posture; the subscript *init* represents the initial value. If we define sensor coordinates at the center of the scanning sonar header shown in Figure 12, the end of the leg with respect to the sensor can be written as follows:

(3) $${}^{S}T_{i4}={\;}^{S}T_{B}{\;}^{B}T_{i4}={\left({}^{G}T_{S}^{p}{\;}^{S}T_{R}^{init}\;R_{ypr}{\left({}^{S}T_{R}^{init}\right)}^{-1}\right)}^{-1}{\;}^{G}T_{S}^{init}{\;}^{S}T_{i4}^{init}$$

With the desired tilting angle displacement of the sensor head, the transformation matrix from the body coordinates to the end of the leg can be obtained by Equation (4). By controlling the body posture displacement with respect to the end of the legs, we get the desired posture displacement of the sensor head with respect to the fixed (ground) coordinates.

(4) $${}^{B}T_{i4}={\;}^{B}T_{S}{\left({}^{G}T_{S}^{p}{\;}^{S}T_{R}^{init}\;R_{ypr}{\left({}^{S}T_{R}^{init}\right)}^{-1}\right)}^{-1}{\;}^{G}T_{S}^{init}{\;}^{S}T_{i4}^{init}$$

**Figure 13:**
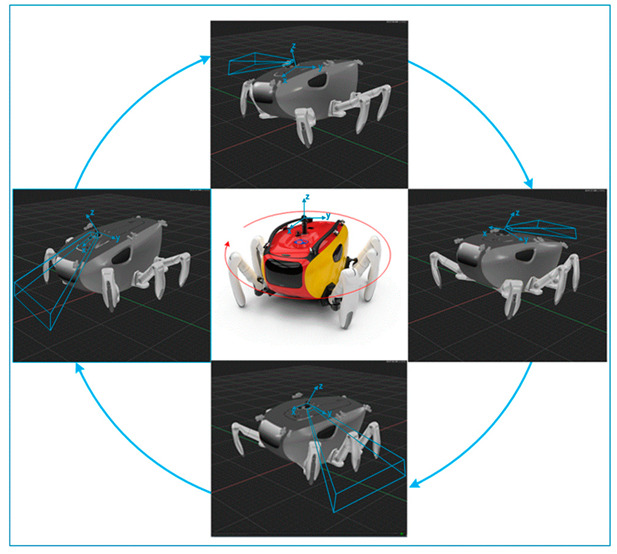
Simulation results of the downward HHM [32].

Figure 13 shows the results of a downward HHM simulation toward the center of the sensor head in the direction the sector-scanning sonar is scanning. We performed a water tank test to confirm the simulation results before the sea trial. This test was conducted in a facility at the KRISO (Korea Research and Institute of Ship and Ocean Engineering) and sonar images were acquired through the downward HHM function [31].

## 4. Review of the Results of Acquisition Data from Sea Trial

**Figure 14:**
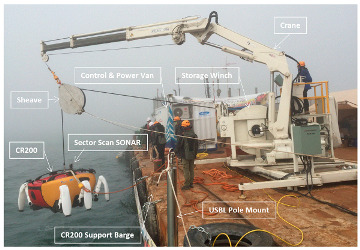
CR200 system on the barge.

**Figure 15:**
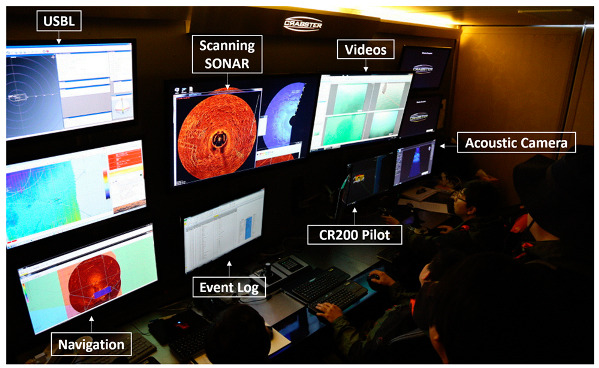
Inside of the control and power van.

In this section, we describe the sea trial and its results to verify the CR200 function discussed in Section 3.1. The sea trial was conducted from 16 April to 15 May, 2015 [33], in the Mado area, located in the West Sea of Korea, with a water depth of 4 to 6 m. Figure 14 and Figure 15 show the support barge and control room of the CR200. The size of the barge was 34 m by 14 m, and was capable of accommodating a crane for the launch and recovery of the CR200 and housing the control room. Because the region was an area where artifacts were being excavated, we randomly placed samples for testing and conducted a mission to explore and collect them.

### 4.1. Analysis of Acquired Sonar Images

**Figure 16:**
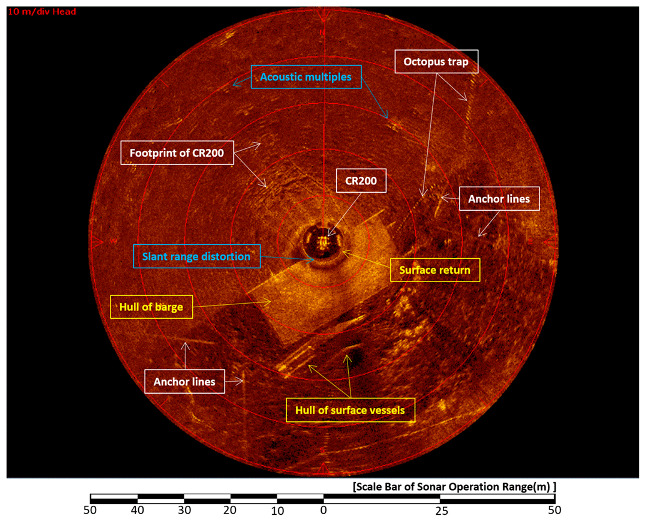
Sonar image of the CR200 in the first sea trial [32].

Figure 16 shows a sector-scanning sonar image obtained in a horizontal position on 30 April, 2015. The sonar was run at a 50-meter operation range, a height of 1.6 m, and a step size of 0.225°. As seen in Section 2, the structure on the sea surface and object in the seabed overlapped because of the shallow water depth. In the center of the image, the top of the CR200 is shown and, at 6 o’clock, the bottom of the support barge and two boats moored to the barge appeared. Other objects in the image are the four anchor lines of the barge and an octopus trap at 2 o’clock; a trace of the CR200 on the seabed can also be seen.

### 4.2. HHM Effect of CR200

**Figure 17:**
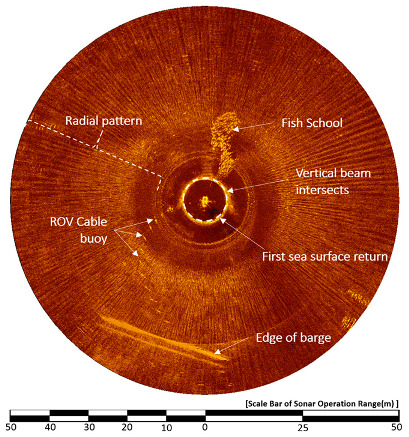
Result image of the 10° upward HHM.

**Figure 18:**
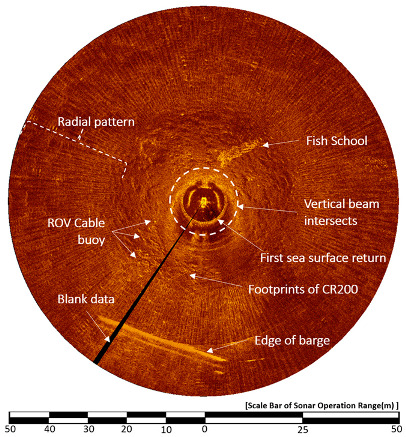
Result image of the horizontal posture.

**Figure 19:**
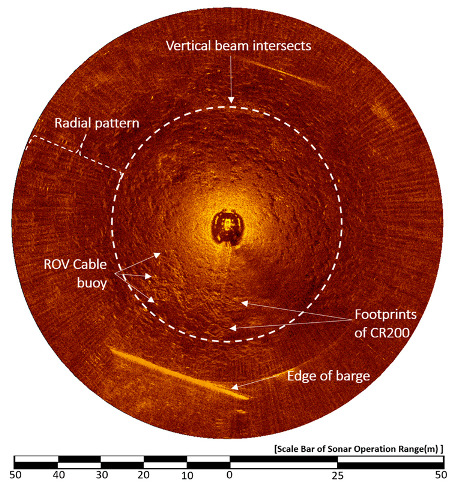
Result image of the 10° downward HHM.

Figure 17, Figure 18 and Figure 19 are sonar images obtained from the same location on 1 May, 2015. Figure 17 shows the result of the CR200 continuously applying an upward 10° HHM in the sonar head scanning direction during the sector-scanning sonar operation. The sonar was run with at a 50-meter operation range, a height of 1.6 m, a step size of 0.225°, and a water depth of 4 to 5 m. The radius of the vertical beam intersection is 5.14 m, as calculated by Equation (1), considering the water depth as 4 m; this is indicated by a white dotted line on the image. The first sea surface return appeared as a strong reflected signal, almost overlapping the white dotted line shown in the vertical beam intersection radius. The radial pattern, one of the sea surface reverberation noises, is generated by acoustic refraction [22]. With this radial pattern, the presence or absence of sea surface reverberation noise can be easily distinguished with the naked eye. The range of the radius is displayed at 10 o’clock, and the sea bottom reverberation image is hardly seen in most areas due to the radial pattern. From 6 to 8 o’clock, the ROV cable buoy on the surface and edge of the support barge can be seen, and 12 to 1 o’clock shows a school of fish, which was detected by volume reverberation noise.

Figure 17 presents another sonar image that the CR200 acquired in the horizontal position. The vertical beam intersections appear from a radius of 8.95, calculated from a depth of 4 m according to Equation (1). A footprint of CR200 can be seen, which was not seen in Figure 16, and at 1 to 2 o’clock, a fish school, the volume reverberation, appears as well. The cable buoy of the ROV faded but was seen clearly in Figure 16.

Figure 19 shows the result of the CR200 continuously applying a downward 10° HHM in the sonar head scanning direction during the sector-scanning sonar operation; conditions were the same as Figure 17. According to Equation (1), the vertical beam intersection is 27.42 m at a water depth of 4 m, which is 18.47 m larger than when upward at 10° HHM. The radial pattern noise discerned by the naked eye was significantly reduced. In addition, the image of the seabed, which is bottom reverberation, appears in detail, showing the footprint of the CR200 right down the barge.

### 4.3. Decreases Slant Range Distortion

**Figure 20:**
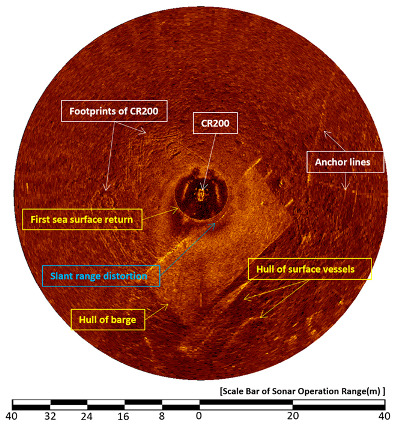
Horizontal sonar image under the support barge.

**Figure 21:**
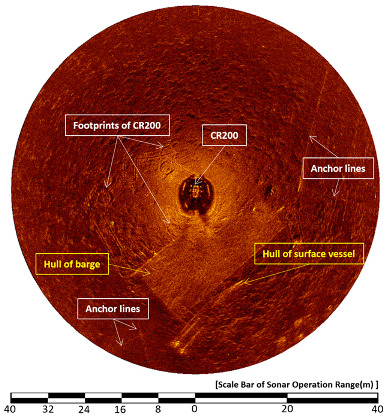
The 6° downward HHM sonar image under the support barge.

Figure 20 and Figure 21 show images acquired on 1 May 2015, and illustrate the slant range distortion noise. The sonar was run at a 40-meter operation range, a height of 1.6 m, a step size of 0.225°, and at a water depth of 4 m. Since the bottom of the barge was located 0.8 m below the water level, it was quite close to the top of the sector-scanning sonar head. The distance between them was 1.6 m. The objects marked in white boxes were seen on the seafloor and are the footprints of CR200, the CR200 itself, and the mooring lines of the barge anchor; whereas the objects marked in yellow boxes were seen on the sea surface, they are the first sea-surface return signal, the hull of the barge, and other boats. The blue boxes mark deformation caused by slant range distortion.

Figure 21 shows the result of the CR200 continuously applying downward 6° HHM in the sonar head scanning direction during the sector-scanning sonar operation; conditions were the same as Figure 20. The first sea surface return image shown in Figure 20 disappeared and the footprints of CR200 are seen right under the barge. The image of a hull of a boat on the surface at 6 o’clock that was previously seen has also disappeared. This confirms that the HHM function is effective in avoiding slant range distortion caused by an object close to the sensor head of the CR200.

### 4.4. Image Synthesis Technique Using Differential Global Positioning System (DGPS)

**Figure 22:**
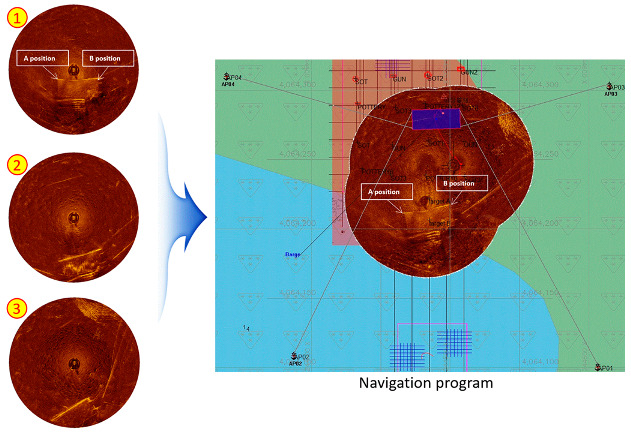
Application of the sonar mosaic image using the differential global positioning system (DGPS) information [32].

As described in Section 2.2, when a sector-scanning sonar survey is performed using a general ROV, it is difficult to acquire an image for the entire 360-degree direction, and there is a limit in maintaining the set direction and proceeding toward the target. Figure 22 shows the results of the exploration of the seabed, based on the position of the differential global positioning system (DGPS) of the barge while the CR200 accumulated a detailed image of the seafloor, acquired by the downward HHM at a shallow water depth. Figure 1 shows the first step to calculate the relative position of the CR200 using DGPS information for the A and B positions of the bottom of the barge. The next step was to synthesize images 2 and 3 one by one, and it was possible to operate in real-time, as shown in the screenshot of the Hypack’s navigation program. This operation suggests that it is possible to accurately measure the location of the seabed or the object on the seabed using only DGPS information of the underwater structure without an additional underwater location tracking system. Considering the deepwater depth case, both upward and downward HHMs were acquired at a fixed position; the former applies to the calculation of underwater sonar center positions, and the latter applies to the collection of submarine information. In this way, location measurements based on the DGPS information are possible in deep water.

### 4.5. Analysis of Experimental Results

We analyzed the difference between the effects of the upward, horizontal, and downward HHM using Equation (1), and Table 3 displays the results. The vertical beam size of the sonar used in the analysis was calculated to be 30°, and it was assumed that there was no side lobe effect. Table 3 shows the range of the first intersection and sea surface reverberation area at an operating range of 4 to 10 m water depth. When the sea surface reverberation area is 100% in the horizontal state, the ratio of the sea surface reverberation area to the HHM inclination angle is shown. The sensor operating radius used in the calculation of the table is 50 m, and the operating depth was the value obtained by subtracting the sensor head altitude value of 1.6 m. In particular, the multipath noise was generated when a range of the first intersection exists within the first half of the sonar operation range, as detailed in Section 2.2. It can eliminate multipath and reduce sea surface reverberation by having it exist at a distance of more than 25 m. This is a fundamental improvement of the sonar image.

For a more specific example, when the sonar is being operated at a 4 m water depth in a horizontal attitude, the sea surface reverberation presents from a radius of 8.95 m. When this area is assumed to be 100%, the sea surface reverberation area of 28% is reduced for images acquired by applying the 10° downward HHM to avoid multipath. 

**Figure 23:**
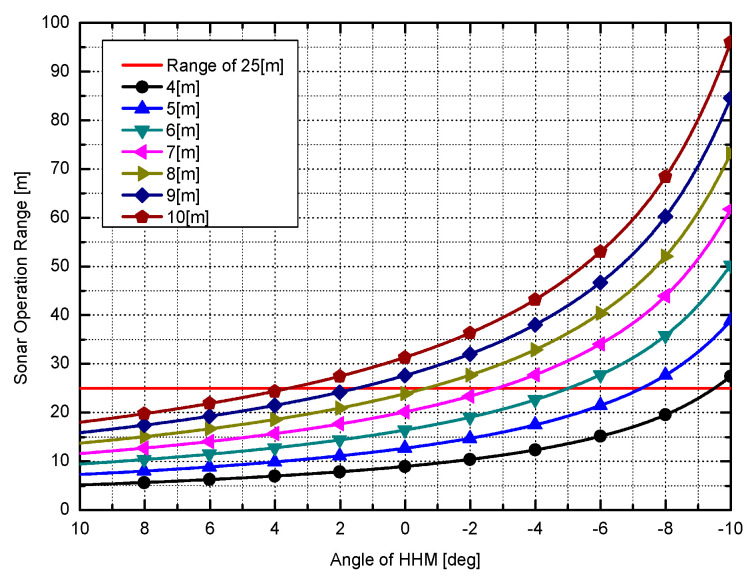
Correlation between the angle of HHM and sonar operation range.

Figure 23 visually shows the values in Table 3. The X-axis is the angle of HHM and the Y-axis is the range of the first intersection. The 25-meter range is marked in red, and multipath noise was removed by selecting the angle of the HHM above the 25-meter crossing point in each depth graph.

**Figure 24:**
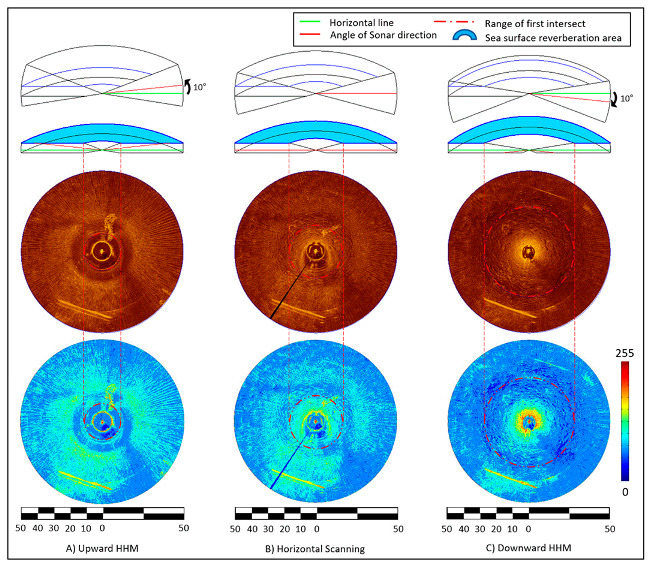
Comparison of scanning images and diagrams of surface reverberation zones according to each HHM [32].

Figure 24 shows the sonar images obtained from the experiments in Section 4, and the applied 10° upward HHM, horizontal, and 10° downward HHM to the sonar from the left, respectively. Using the AutoCAD program, the interference area of the water surface was displayed as a blue donut on the top of the sonar image, and the sonar scanning angle was represented by a red line. The blue background image at the bottom shows the brightness of the sonar pixels divided into 0 to 255 color distributions. A radial pattern noise on the surface of the water can be seen, and it was also confirmed that the red color with the highest color intensity was concentrated on the seabed near the CR200 in the image with the 10° downward HHM applied (right image). It shows the improvement effect of the sonar image for the three different postures.

## 5. Conclusions

In this study, we proposed several strategies for use with the CR200 to acquire precise and efficient images through both the HHM and the control posture function of the CR200. To verify the strategies suggested, a sea trial was conducted to acquire the sonar image, and by analyzing the results from the sea trial, a positive result was confirmed. 

Two main effects can be confirmed through the HHM function. First, it is a technology for selectively separating sea surface reverberation and sea bottom reverberation. If you want to see sea bottom reverberation, applying a downward HHM can reduce sea surface reverberation noise, multipath noise, and minimize slant range distortion. Second, the CR200 was able to acquire 360° omnidirectional images with one sector scanning sonar. Moreover, by applying the downward HHM, it was possible to minimize the shadow area and increase image acquisition efficiency.

The control posture function is a technology that provides attitude and position to the sonar under four environmental conditions (shallow water, regions with a strong current, an uneven sea bottom, or a sloped sea bottom) and in Section 3.3, a previous study was introduced to replace the detailed explanation. This is a technology that allows the platform to move to the desired course while maintaining a certain altitude and heading. 

The next study will be to improve the accuracy and efficiency of sensor data by linking the robot’s degrees of freedom to various marine measurement sensors. The technology described in this study is expected to be highly useful for mine search and identification in shallow water, the separation and acquisition of seabed and ice shelf data in polar exploration, detailed investigation of offshore structures, and search and investigation of wrecks and cultural heritage.

## Figures and Tables

**Table 1:** Specifications of sector-scanning sonar.

Model	1171 High-Resolution Scanning Sonar	
Depth rating	3000 m	
Frequency	675 kHz	
Beam width	0.9° × 30°~ Fan	1.7° × 1.7° Cone
Range	0.5–100 m	
Range resolution	≥19 mm	
Scan speed	11 s/360° @ 10 m & 1.8° step 36 s/360° @ 100 m & 1.8° step	
Scan Width	360° continuous	
Step size	≥0.225°	
Pulse lengths	25–2500 µs	
Power	33 W, 22–60 VDC (voltage direct current)	

**Table 2:** Specification of the CR200.

Parameter	Value
Size [m]	2.42 (L) × 2.45 (W) × 1.16 (H)
Weight [Kg] Frame weight [Kg]	682 (air)/188 (water) 62
Max. ground clearance [m]	Over 0.5
Number of legs	6
Max. walking speed [m/s]	0.5
Max. depth of water [m]	200
Max. endurable current [m/s]	3
Power supply [V]	150–190 (DC)

**Table 3:** Correlation between the angle of HHM and sonar operation range.

Motion		Upward HHM					Horizontal	Downward HHM				
Degree		10	8	6	4	2	0	2	4	6	8	10
4 m	Range of first intersect (m)	5.14	5.65	6.25	6.97	7.85	8.95	10.39	12.34	15.15	19.54	27.42
	Sea surface reverberation area (m^2^)	7766	7749	7727	7697	7656	7597	7510	7129	6650	5487	1262
	Percentage of Area (%)	102	101	101	101	100	100	98	97	93	87	72
5 m	Range of first intersect (m)	7.29	8.00	8.85	9.87	11.12	12.69	14.72	17.48	21.46	27.68	38.85
	Sea surface reverberation area (m^2^)	7683	7648	7603	7543	7461	7344	7169	6889	6403	5442	3108
	Percentage of Area (%)	104	104	103	102	101	100	97	93	87	74	42
6 m	Range of first intersect (m)	9.43	10.36	11.46	12.77	14.39	16.42	19.05	22.63	27.77	35.83	50.28
	Sea surface reverberation area (m^2^)	7570	7512	7437	7337	7199	7002	6709	6241	5427	3818	−89
	Percentage of Area (%)	108	107	106	104	102	100	95	89	77	54	−1
7 m	Range of first intersect (m)	11.58	12.72	14.06	15.68	17.66	20.15	23.38	27.77	34.09	43.97	61.71
	Sea surface reverberation area (m^2^)	7428	7341	7228	7077	6870	6574	6132	5427	4200	1778	−4109
	Percentage of Area (%)	122	121	119	116	113	100	101	89	69	29	−67
8 m	Range of first intersect (m)	13.72	15.07	16.67	18.58	20.93	23.88	27.71	32.92	40.40	52.11	73.14
	Sea surface reverberation area (m^2^)	7258	7136	6977	6765	6473	6057	5437	555+	2723	−678	−8948
	Percentage of Area (%)	119	117	115	111	106	100	89	73	44	−11	−147
9 m	Range of first intersect (m)	15.86	17.43	19.27	21.49	24.20	27.62	32.04	38.06	46.71	60.26	84.57
	Sea surface reverberation area (m^2^)	7059	6895	6683	6399	6010	5454	4624	3300	996	−3552	−14608
	Percentage of Area (%)	116	113	110	105	99	100	76	54	16	−58	−241
10 m	Range of first intersect (m)	18.01	19.78	21.88	24.39	27.47	31.35	36.37	43.20	53.03	68.40	96
	Sea surface reverberation area (m^2^)	6831	6620	6346	5980	5479	4762	3694	1987	−980	−6842	−21088
	Percentage of Area (%)	143	138	133	125	115	100	77	41	−20	−143	−442
